# Acute Idiopathic Ulceration of Cartilage

**Published:** 1849-12

**Authors:** Theophilus Mack

**Affiliations:** St. Catharines, C. W.


ART. IV.—*Acute Idiopathic Ulceration of Cartilage.* By Theophilus Mack, M. D., of St. Catharines, C. W.

The following is a brief and imperfect sketch of a case presenting the characteristic symptoms of the above disease, which occurred in the person of a young girl in her fifteenth year. Upon my first visit, Sept. 18th, 1848, I found my patient tossing about on the bed, and screaming aloud occasionally, from excruciating pain experienced in the knee joint of the right limb, with which, she stated, she had been seized shortly after her return from a long walk, about forty-eight hours before my arrival.

The pain, ere it had fixed upon the joint, developed itself in the temporo maxillary articulation; next in the left shoulder, thence it metastased to the right hip, and, finally, established itself in the knee of the same side. The symptoms, upon examination, were, a clear, delicate skin, light hair, pearly conjunctiva, thick lips, cervical glands large, and other evidences of a strongly marked, strumous diathesis.

She was excessively neuropathic, complaining of pain on pressure at various points; face flushed, skin hot and dry, tongue white, bowels constipated; pulse 130, quick and tense. No abdominal or thoracic lesion diagnosed. The menstrual discharge had not yet been established. She now appeared to me to be laboring under acute articular rheumatism, except that no *tumor*, or *tension*, or *redness* of surface appeared in the inflamed joint. The pain was mitigated also by *extending* the leg, thus diminishing the pressure of the inflamed articular surfaces, and I found that her mother had frequently, as she expressed it, "pulled the knee," to afford her relief. The least flexion of the joint occasioned intolerable suffering. Ft. v. s. ad ℥ xx, ℞. hyd. chlorid., pulv. rhei, aa., gr. x., ft, pulv. statim sumend; etiam ℞. antim. pot. tart. gr. ii.; tinct. aconiti ʒ ss., aquæ f. ℥ iv., ft. mist. cujus capiat ℥ ss. 3 tiis horis.

19th.—Bowels freely purged. Urgency of symptoms continues. Repetat v. s. ℞. hyd. chlorid. gr. viij., pulv. ipecac. comp. ʒ i., m., in part. æquales viij. distribuend. capt. tertia quaque hora; contin. mist. ut heri.

20th.—Pain of joint undiminished, febrile symptoms subdued; cucurbit. cruent. a vicinia artus. ℞. hyd. chlorid. gr. viij. morphiæ hydrochlor. grs. iv. m. et in chartul. viij. divide, quarum sumatur i quadrihorio; mist. ut heri.

21st.—The local pain has abated in a great measure, being now only felt upon motion. Some swelling in the tissues about the joint. Perfect rest and fomentations were directed. Omitte misturam, contin. pulv.

22d.—Ut supra. Omitte pulv.

23d.—Pain paroxysmal, at long intervals, and recurring upon motion. Diarrhœa; mist., cret., comp., ℥ ii. 3 tiis horis.

24th.—An unhealthy mercurial action has supervened, aphthæ appearing on the fauces, and diarrhœa.

25th.—No swelling or fluctuation in the joint; some œdema of the parts adjacent. The diarrhœa has yielded to anodyne enemata, and small doses of Dover's powder.

26th.—Pain still felt upon motion in the knee, but of a much less acute nature than heretofore. Much to my dissatisfaction, her mother now became impressed with the opinion that she was convalescent, or sufficiently so to dispense with my attendance, and fearful of the imputation of an "itching palm," I refrained from remonstrating, and took my departure, after prescribing small doses of iodide of potassium three times a day, and counter-irritation in the neighborhood of the joint. Eight days after, I was again summoned, in the middle of the night, to her bedside. I found the parts about the diseased knee and leg tender, swollen and œdematous fluctuation being discovered, I made a puncture at the point where the fluid appeared about to effect its escape, viz., about the middle of the spine of the tibia; this was followed by a large discharge of laudable pus. Great temporary relief ensued.

October 2d.—In consultation with Dr. Telfer, of Toronto, it was decided from the circumstance of the suppuration being *external* to the joint, the character of the pus, &c., to postpone operative interference. A probe passed along the sinus, leading to the opening, reached the head of the tibia. Another deep incision in a depending position, was practised near the joint, but the pus still preferred the original route. Porter and nourishing diet. ℞. quiniæ disulph. gr. ii., tertia q. q. h. sumend.

During several successive days, five incisions were made for the liberation of pus, in different parts of the leg; one near the ankle. From this latter, issued much thin discharge of purulent matter, containing small masses resembling *lumps of curd*.

Discoloration now appearing about the sacrum and trochanter, she was placed upon an air bed, but large sloughs soon began to separate, and an hydrostatic bed was procured.

Nov. 23d.—In consultation with Dr. Telfer. Symptoms of hectic; skin moist; pulse 100, soft; tongue smooth; a few aphthæ still remain upon the velum palati; extremities œdematous; large healthy looking ulcers over the sacrum and trochanter; ascribes to the water bed the most grateful relief. The discharge from the incisions thin, sanious, and mingled with curdlike masses; the probe passed along the surface of the tibia, communicates the rough sensation of diseased bone. Our poor patient's condition was now deplorable; the discharges from the diseased leg, would, alone, have proved most exhausting, but the bed sores, one about six inches in circumference, added to her accumulated misery. There was not so much emaciation as might have been expected; no symptoms of thoracic lesion could be detected, and, as the constitutional powers appeared to warrant an operation, immediate amputation above the knee was decided upon.

The following morning, assisted by Drs. Telfer and Rolls, I proceeded to the operation. The consequent loss of blood did not exceed ℥ iv. She sustained the shock remarkably well. Appearances on dissection of amputated member:—The muscular tissues were very indistinct, and surrounded, in many situations, by collections of pus. No pus detected in the cavity of the knee joint; the synovial membrane appeared slightly vascular; the semilunar cartilages were completely absorbed, and the ulceration had destroyed every vestige of structure of this nature, about the head of the tibia, which partook of the disease also, and was perforated by a sinus, affording a communication between the joint and the cavity of an abscess over the spine of the tibia. The whole shaft and malleolar extremity of this bone were extensively carious, and exfoliating in some places. A large ulcerated surface, exposing the bony structure beneath, occupied each condyle of the femur, over the inferior two-thirds of their superficies. A small ulcerated spot also appeared on the anterior portion of the cartilage of the outer condyle. On each side of the vertical line of the patella, the cartilages were ulcerated. The disease presented the same appearances in both tibio-fibular articulations, and traces of it were detected in the cartilages of the ankle joint.

Fourteen days after the operation, the ligatures came away. Part of the stump had healed by the adhesive process, and she appeared to be *doing well.* With the exception of the smooth tongue, and slight abdominal tenderness, no alarming symptoms presented. In a few days hectic supervened, and rale crepitant in both subclavicular regions, hæmoptysis and diarrhœa. The discharges from the stump ceased, and dyspnœa rapidly increased. She soon succumbed to acute tuberculosis, and died on the 24th day after the amputation, and nearly three months from the invasion of the disease. No post-mortem allowed. The rapidity with which so large a quantity of bone arrived at an advanced stage of disease, (in sixty-six days) and the reputed rarity of acute ulceration of cartilage, from purely constitutional causes, with the distinct absence of synovitis, have induced me to submit the above for publication, not so much from being afflicted with the *cacoethes scribendi*, as from the desire to be numbered among those commended by St. Bernard:—"Sed sunt quoque qui scire volunt ut ædificent, et charitas est. Et item qui scire volunt ut ædificentur, et prudentia est."

